# Sitravatinib as a potent FLT3 inhibitor can overcome gilteritinib resistance in acute myeloid leukemia

**DOI:** 10.1186/s40364-022-00447-4

**Published:** 2023-01-24

**Authors:** Yvyin Zhang, Peihong Wang, Yang Wang, Yang Shen

**Affiliations:** 1 Shanghai Institute of Hematology, State Key Laboratory of Medical Genomics, National Research Center for Translational Medicine (Shanghai), Ruijin Hospital Affiliated to Shanghai Jiao Tong University School of Medicine, Shanghai, 200025 China; 2 Department of Hematology, Guangzhou First People’s Hospital, School of Medicine, South China University of Technology, Guangzhou, 510000 China

**Keywords:** Sitravatinib, *FLT3*-ITD, AML, Gilteritinib, Drug resistance, *FLT3*-ITD-F691L, FL, FGF2

## Abstract

**Background:**

Gilteritinib is the only drug approved as monotherapy for acute myeloid leukemia (AML) patients harboring FMS-like tyrosine kinase 3 internal tandem duplication (*FLT3*-ITD) mutation throughout the world. However, drug resistance inevitably develops in clinical. Sitravatinib is a multi-kinase inhibitor under evaluation in clinical trials of various solid tumors. In this study, we explored the antitumor activity of sitravatinib against *FLT3*-ITD and clinically-relevant drug resistance in *FLT3* mutant AML.

**Methods:**

Growth inhibitory assays were performed in AML cell lines and BaF3 cells expressing various *FLT3* mutants to evaluate the antitumor activity of sitravatinib in vitro. Immunoblotting was used to examine the activity of FLT3 and its downstream pathways. Molecular docking was performed to predict the binding sites of FLT3 to sitravatinib. The survival benefit of sitravatinib in vivo was assessed in MOLM13 xenograft mouse models and mouse models of transformed BaF3 cells harboring different *FLT3* mutants. Primary patient samples and a patient-derived xenograft (PDX) model were also used to determine the efficacy of sitravatinib.

**Results:**

Sitravatinib inhibited cell proliferation, induced cell cycle arrest and apoptosis in *FLT3*-ITD AML cell lines. In vivo studies showed that sitravatinib exhibited a better therapeutic effect than gilteritinib in MOLM13 xenograft model and BaF3-*FLT3*-ITD model. Unlike gilteritinib, the predicted binding sites of sitravatinib to FLT3 did not include F691 residue. Sitravatinib displayed a potent inhibitory effect on *FLT3*-ITD-F691L mutation which conferred resistance to gilteritinib and all other FLT3 inhibitors available, both in vitro and in vivo. Compared with gilteritinib, sitravatinib retained effective activity against *FLT3* mutation in the presence of cytokines through the more potent and steady inhibition of p-ERK and p-AKT. Furthermore, patient blasts harboring *FLT3*-ITD were more sensitive to sitravatinib than to gilteritinib in vitro and in the PDX model.

**Conclusions:**

Our study reveals the potential therapeutic role of sitravatinib in *FLT3* mutant AML and provides an alternative inhibitor for the treatment of AML patients who are resistant to current FLT3 inhibitors.

**Supplementary Information:**

The online version contains supplementary material available at 10.1186/s40364-022-00447-4.

## Background

Activating mutations of FMS-like tyrosine kinase 3 (*FLT3*) are among the most frequently detected genetic alterations in acute myeloid leukemia (AML), accounting for approximately 30–35% of all patients [1, 2]. There are two canonical types of *FLT3* mutations: internal tandem duplication within the juxta-membrane domain (*FLT3*-ITD) found in 25–30% of AML patients and point mutations in the tyrosine kinase domain (*FLT3*-TKD) in 5–7% of cases [1, 2]. These mutations can ligand-independently and constitutively activate FLT3 and its downstream proliferative signaling cascades involving STAT5, PI3K/AKT and RAS/MAPK, resulting in the expansion of leukemic cells [3, 4]. *FLT3*-ITD mutations are clinically associated with high white blood cell counts, increased risk of relapse and poor overall survival (OS), making it a promising therapeutic target in AML [1, 5–7].

Various FLT3 tyrosine kinase inhibitors (TKI) have been developed over the past decades. The first‐generation FLT3 inhibitors including tandutinib, sunitinib, midostaurin, lestaurtinib and sorafenib were not originally designed to target FLT3 kinase and their clinical efficacy was limited by modest inhibition of FLT3 as well as development of drug resistance. Thus the second-generation FLT3 inhibitors with greater anti-FLT3 efficacy such as gilteritinib, quizartinib, and crenolanib, were developed [8–10]. To date, many FLT3 inhibitors have been evaluated in clinical trials, yet gilteritinib is the only drug that has been successfully approved worldwide as a monotherapy for the treatment of AML patients harboring *FLT3* mutations. Gilteritinib significantly improved median overall survival than salvage chemotherapy (9.3 *vs.* 5.6 months) for refractory/relapsed patients. However, approximately one third of patients had no response and only 37% of patients survived more than one year due to the emergence of drug resistance [11].

The mechanisms of drug resistance to FLT3 TKI can be classified as either primary or secondary [12]. Growth factors and cytokines in bone marrow (BM) microenvironment supporting the survival of residual leukemia cells during FLT3 inhibitor treatment confer primary drug resistance [13, 14]. It has been demonstrated that FLT3 ligand (FL) and fibroblast growth factor 2 (FGF2) catalyze temporal evolution of early to late gilteritinib resistance through persistent activation of MAPK and AKT pathways [15]. Secondary resistance involves acquired on-target TKD mutations and off-target mutations of other genes. Acquisition of *FLT3*-TKD mutations at D835, Y842, F691 or other sites is the most common mechanism of resistance against type II FLT3 inhibitors that bind inactive FLT3 [16, 17]. Gilteritinib as a Type I FLT3 inhibitor which interacts with the ATP-binding site can overcome D835 and Y842 mutations. However, gilteritinib-treated patients acquired the gatekeeper mutation F691L at relapse and there are no available FLT3 inhibitors for patients with F691L mutation [18, 19]. Resistance mutations can also occur in off-target genes that rend leukemic cells independent of FLT3 signaling, as exemplified to *IDH1/2*, *TP53*, *TET2* [20]. Accordingly, developing new FLT3 inhibitors, especially those able to overcome gilteritinib resistance, is of unmet clinical demand.

Sitravatinib is a novel potent broad-spectrum TKI with immunomodulatory effects, which can block the phosphorylation of PDGFRα, PDGFRβ, IGF1-R and c-Met [21, 22]. It has exerted effective antitumor activity and good safety in clinical trials of various solid tumors such as sarcoma, breast cancer and clear cell renal carcinoma [23–25]. No studies have yet assessed the efficacy of sitravatinib in hematologic malignancies. Here, we identified sitravatinib as a FLT3 inhibitor and tested the therapeutic effects of sitravatinib in AML using in vitro and in vivo models. Our preclinical study demonstrates that sitravatinib is a potent FLT3 TKI and can overcome clinically-relevant gilteritinib resistance mediated by F691L mutation or increased FL/FGF2, which may represent a promising second-line TKI for AML patients harboring *FLT3* mutations.

## Methods

### Chemicals and reagents

Sitravatinib, gilteritinib and quizartinib were purchased from TargetMol (Boston, USA). For in vitro experiments, all the drugs were reconstituted in DMSO to 10 mM for storage and further diluted in the culture medium with the final concentration of DMSO below 0.1%. For in vivo animal experiments, gilteritinib and quizartinib were suspended in a 0.5% methylcellulose solution and sitravatinib was dissolved in a formula containing 5% DMSO, 30% PEG300, 10% Tween 80 and 55% sterile water. DMSO, methylcellulose, PEG300, Tween 80 and busulfan were provided by MedChemExpress (Monmouth Junction, USA). FL and FGF2 were provided by PeproTech (Cranbury, USA).

### Cell lines

The human leukemia cell lines used were purchased from the American Type Culture Collection (Manassas, USA). BaF3 cell lines stably expressing human *FLT3* with different mutations were generated as previously described [26]. Cells were cultured in RPMI-1640 (BasalMedia, Shanghai, China) supplemented with 10% FBS (Gemini, USA), 100 U/mL penicillin (Yeasen, Shanghai, China), and 50 mg/ml streptomycin (Yeasen) and maintained in a humidified chamber delivering 5% CO2 at 37 °C. BaF3 cells also required 3 ng/ml IL-3 (R&D Systems, Minneapolis, USA) to support survival.

### Cell viability assay

Cells (5–10 × 10^3^ cells/100uL/well) were seeded into 96-well plates and treated with the indicated concentrations of the corresponding drug in triplicate. After 48 h, cellular proliferation was assessed by CellTiter-Glo® 2.0 Cell Viability Assay (Promega, Madison, USA) according to the manufacturer’s instructions and luminescence was measured using Varioskan Flash (ThermoFisher, Waltham, USA). Dose–response curves were generated and the 50% inhibitory concentration (IC50) was determined with GraphPad Prism.

### Cell apoptosis and cell cycle assay

MV4-11, MOLM13 cells (2.0 × 10^5^ cells/2 mL/well) were planted in 6-well plates and incubated with increasing concentrations of gilteritinib or sitravatinib for 24 h or 48 h as designed. For cell apoptosis analysis, cells were stained with Annexin V/propidium iodide (PI) Apoptosis Detection Kit (Invitrogen, Carlsbad, USA) and analyzed by flow cytometry (BD LSRFortessa, Franklin Lakes, USA). For cell cycle analysis, the cells were harvested after 24-h treatment and fixed in 70% ethanol overnight. Then, samples were labeled with PI (Biolegend, San Diego, USA) and flow cytometry was performed to detect DNA contents. The results were analyzed using Flowjo software version10.

### Immunoblotting

Western blot analysis was performed as previously described [27]. Briefly, cells after treatment were washed in PBS (BasalMedia, Shanghai, China) and lysed using Laemmli 2 × concentrate sample buffer (Sigma-Aldrich, Saint Louis, USA). After centrifugation, soluble protein fractions were separated onto 8%-12% SDS-PAGE gels and transferred to a nitrocellulose membrane (Millipore, Darmstadt, Germany). The films were incubated with the following primary antibody at 4 °C overnight: anti-Phospho-FLT3 (Tyr589/591) (#3464, CST, Danvers, USA), anti-FLT3/CD135 (ab245116, Abcam, Cambridge, UK), anti-Phospho-STAT5 (Tyr694) (#9359, CST), anti-STAT5A/B (A5029, ABclonal, Wuhan, China), anti-Phospho-AKT-S473 (AP1208, ABclonal), anti-Pan-AKT (A18675, ABclonal), anti-Phospho-ERK1/2 (Thr202/Tyr204) (#4370, CST), anti-ERK1/2 (#4695, CST), anti-Caspase8(13423–1-AP, Proteintech, Rosemont, USA), anti-PARP1 (#13371–1-AP, Proteintech), HRP-conjugated Alpha Tubulin (#HRP-66031, Proteintech). For protein bands detection, the HRP-conjugated anti-rabbit secondary antibody (#7074, CST) was incubated for 2 h and the signals were visualized by Immobilon Western HRP Substrate (Millipore, Darmstadt, Germany) in Amersham Imager 600 system (GE Healthcare, Chicago, USA). The densitometry of protein bands was quantified via ImageJ. And the expression of protein was normalized to tubulin and expressed as fold change compared with vehicle control.

### The cellular thermal shift assay

The cellular thermal shift assay (CETSA) was carried out as the previous literature described [28]. In short, the first step was to determine the melting curve. 1 × 10^7^ BaF3-*FLT3*-ITD cells in 10 mL culture medium were incubated with sitravatinib (30 μM/mL) or DMSO for 1 h in a CO2 incubator at 37 °C. Then the cells were resuspended in PBS supplemented with protease inhibitors (TargetMol, Boston, USA) and distributed into 7 different PCR tubes to receive heat treatment under different temperatures as designed for 3 min. The protein was extracted using liquid nitrogen and quantitated by immunoblotting. We chose the temperature at which a majority of FLT3 was almost undetectable in DMSO but was apparent in sitravatinib for isothermal dose–response experiments. 1 × 10^7^ BaF3-*FLT3*-ITD cells in 10 mL culture medium were divided evenly into 10 wells of a 12-well plate and treated with serial concentrations of sitravatinib for 30 min. Next, the samples were transferred to PCR tubes and heated at the appropriate temperature for 3 min. Cell lysis and detection of soluble protein were performed as in the first step. Protein bands were quantified by Image Lab and the data was analyzed by GraphPad Prism.

### Molecular docking

The protein FLT3 (PDB ID: 6JQR) was processed using Schrödinger's Protein Preparation Wizard module. The process was as follows: water of crystallization was removed, missing hydrogen atoms were added, and missing bond information was repaired, missing peptides were repaired, protonated at pH 7.0 ± 2.0, assigned hydrogen bonds at pH 7.0, removed water molecules outside three hydrogen bonds, performed energy optimization under the OPLS_2005 force field, then selected the original eutectic ligand as the docking site in the Grid Generation module to generate a grid file. Protonation of sitravatinib at pH 7.0 ± 2.0 in OPLS_2005 force field was performed using the LigPrep module. XP precision docking in the Liand Docking module was selected to perform molecular docking, and the results were visualized using Discovery Studio 2019 and PyMOL.

### In vivo efficacy studies

Animal experiments were conducted in accordance with the established guidelines and were approved by the Institutional Animal Care and Welfare Committee of Ruijin Hospital affiliated to Shanghai Jiao Tong University School of Medicine. In the MOLM13 tumor xenograft model, five-week-old female NSG mice (Shanghai Model Organisms, Shanghai, China) were intravenously injected 5 × 10^6^ MOLM13 cells (day 0). 1 week after cell inoculation, gilteritinib (30 mg/kg), quizartinib (10 mg/kg), sitravatinib (20 mg/kg) or vehicle was dosed daily by oral gavage for 14 days. The applied doses of gilteritinib and quizartinib were the most commonly used maximum dosages for *FLT3*-ITD-leukemia mice in current studies [29, 30], and the dosage of sitravatinib was determined through preliminary experiments and previous pharmacokinetic studies of solid tumors [22]. According to previous studies, the maximum plasma concentration (Cmax) of sitravatinib after oral administration of 20 mg/kg/day for 14 consecutive days was lower than that of quizartinib after a single dose of 10 mg/kg in nude mice [22, 31]. And the Cmax of sitravatinib was less than that of gilteritinib after the same dose treatment for 15 days in clinical patients. Thus the expected serum level of sitravatinib was no higher than that of gilteritinib and quizartinib at the dosages we applied [32, 33]. 3 mice of each group were sacrificed on day 24 and the percentage of human CD45 positive cells in bone marrow (BM) and spleen (SP) was detected by flow cytometry. The survival time was determined upon signs of distress/disease such as hind-limb paralysis, rough coat, and decreased activity. GraphPad Prism was used to plot Kaplan–Meier survival curves.

In the BaF3 model, 2 × 10^6^ BaF3 cells expressing *FLT3*-ITD or *FLT3*-ITD-TKD were injected into six-week-old female BALB/c mice (Charles River, Beijing, China) via the tail vein (day 0). From the second day, mice were randomized into four groups and received therapy (vehicle, gilteritinib (30 mg/kg), quizartinib (10 mg/kg) or sitravatinib (20 mg/kg)) until the first mouse died in the vehicle group. In order to assess the leukemia burden, peripheral blood (PB) was collected and 3 mice of each group were sacrificed to collect BM and SP cells. In the flow cytometry analysis, leukemia cells were defined as GFP-positive cells. GraphPad Prism was used to plot Kaplan–Meier survival curves.

For patient-derived xenografts, six-week-old female NSG mice (Shanghai Model Organisms, Shanghai, China) were sub-lethally treated with busulfan (30 mg/kg) 24 h before injection of 2 × 10^6^ human AML cells harboring *FLT3*-ITD. Mice were sacrificed when displayed signs of distress/disease and BM cells were collected for secondary transplantation (5 × 10^5^ BM cells per mouse). 40 days after cell injection, gilteritinib (30 mg/kg), quizartinib (10 mg/kg), sitravatinib (20 mg/kg) or vehicle was dosed daily for 21 days. The percentage of human CD45 positive cells in PB was detected by flow cytometry before treatment and 21 days after treatment. When the mice in the vehicle group began to die at day 120, 3 mice of each group were sacrificed and the percentage of human CD45 positive cells in BM and SP was detected by flow cytometry.

### Clinical samples

Primary BM cells from AML patients were obtained from Ruijin Hospital, Shanghai Jiao Tong University School of Medicine. Peripheral blood mononuclear cells (PBMCs) were donated by healthy individuals. This study was approved by the Institutional Review Board of Ruijin Hospital, Shanghai Jiaotong University School of Medicine in accordance with the Declaration of Helsinki. The informed consent was obtained from all patients. Primary cells were purified using Ficoll-Paque Plus (Cytiva, Uppsala, Sweden) density gradient centrifugation and cultured in RPMI-1640 supplemented with 20% FBS. Detailed information of each patient was documented in supplementary Table S1.

### RNA-sequencing

MOLM13 cells were treated with gilteritinib (10 nM) or sitravatinib (10 nM) for 24 h. Total RNA was extracted using AllPrep DNA/RNA Mini Kit (Qiagen, Hilden, Germany). The cDNA library was constructed using KAPA RNA HyperPrep kit (Roche, Wilmington, USA) and was sequenced using an Illumina HiSeq™ sequencing platform (San Diego, USA). R package DESeq2 was employed to identify differential gene expression. Genes with an absolute value of fold change ≥ 2 and *p* < 0.05 were used for subsequent analysis.

### Statistical analysis

GraphPad Prism 8.0 software was used to perform statistical analyses. Differences were analyzed utilizing the paired or unpaired 2-tailed Student’s t-test (*P < 0.05; **P < 0.01; ***P < 0.001, **** *P* < 0.0001). IC50 values were calculated by nonlinear best-fit regression analysis. Kaplan–Meier survival curves were compared via the log-rank test. Error bars represent the mean plus or minus standard error of the mean and the statistical significance level was set at *P* < 0.05.

## Results

### Sitravatinib exerts marked anti-leukemia activities against *FLT3*-ITD AML cells

**Fig. 1:**
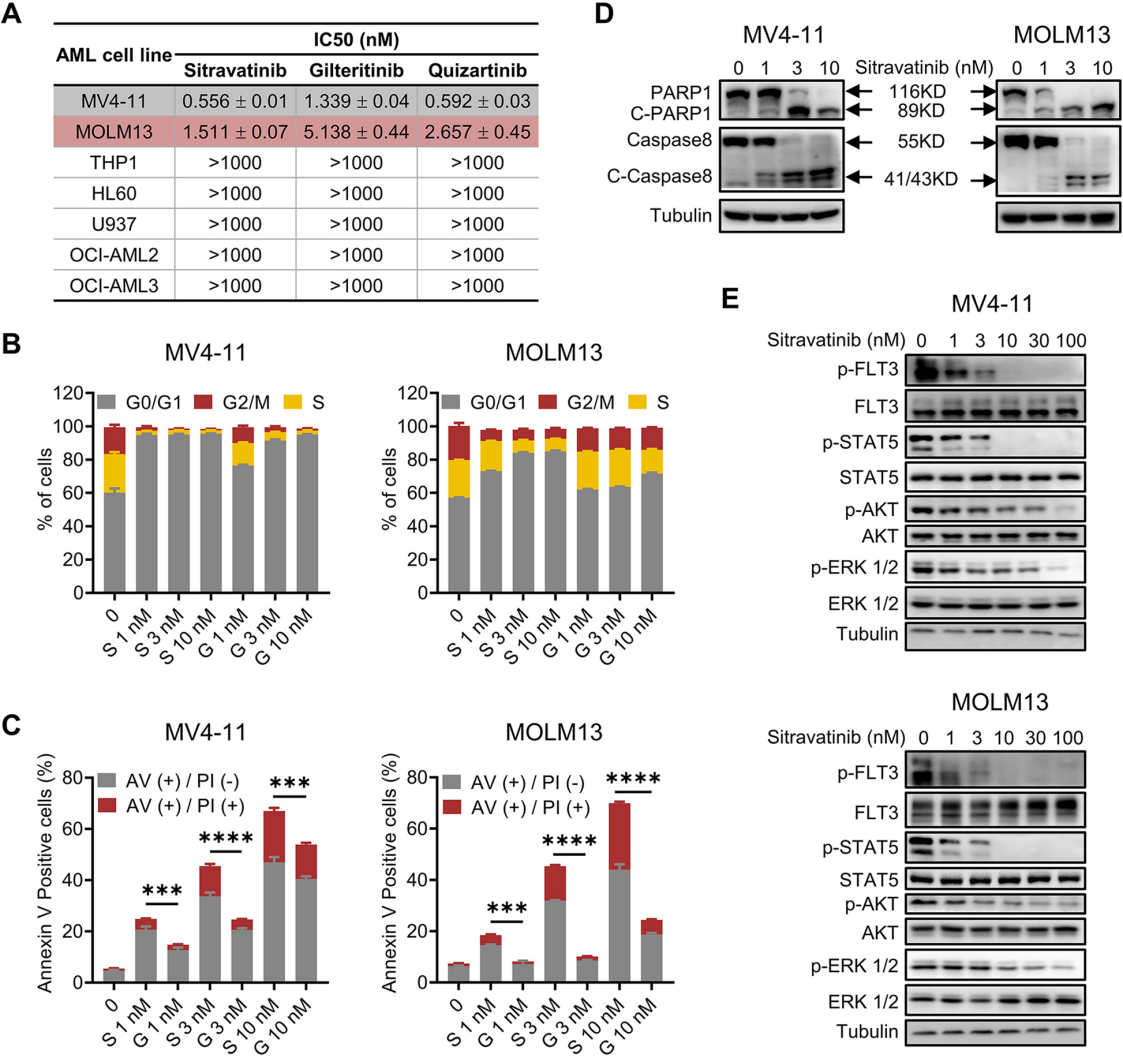
Sitravatinib exerts potent anti-tumor activities against *FLT3*-ITD AML cell lines. **A** The IC50 values of each drug for *FLT3*-WT (THP1, HL60, U937, OCI-AML2, OCI-AML3) or *FLT3*-ITD (MV4-11 and MOLM13) AML cell lines. Data are mean ± standard error from three independent experiments. **B** The percentage of cells in different cell cycle phases after 24-h treatment with various concentrations of sitravatinib (S) or gilteritinib (G). Error bars indicate mean ± standard error, *n* = 3 independent experiments for each cell line. **C** Apoptotic cell populations of MV4-11 and MOLM13 cell lines after 48-h treatment with sitravatinib (S) or gilteritinib (G). Error bars indicate mean ± standard error, *n* = 3 independent experiments for each cell line. ***P < 0.001, ****P < 0.0001. **D** Western blot analysis of PARP1, caspase 8, cleaved-PARP1 (C-PARP1) and cleaved-caspase8 (C-caspase8) expression in MV4–11 and MOLM13 cells after treatment with different doses of sitravatinib for 48 h. **E** Western blot analysis of p-FLT3, p-STAT5, p-AKT and p-ERK 1/2 in MV4-11 and MOLM13 cells after treatment with sitravatinib at the indicated doses for 4 h

To determine the therapeutic potential of sitravatinib against AML, cell proliferation screening was performed on various human leukemia cells (the characteristics of cell lines were shown in Additional file 1: Fig. S1A). *FLT3*-ITD expressing cell lines MV4-11 and MOLM13 were extremely sensitive to sitravatinib with IC50 values of 0.556 nM and 1.511 nM, respectively, while little cytotoxic effect was observed in *FLT3* wild-type (*FLT3*-WT) cell lines (THP1, HL60, U937, OCI-AML2, OCI-AML3) (Fig. 1A, Additional file 1: Fig. S1B). Further IC50 tests showed the growth inhibitory effects of sitravatinib on MV4-11 and MOLM13 were equivalent to that of quizartinib and superior to that of gilteritinib (Fig. 1A, Additional file 1: Fig. S1C, D). Cell cycle analysis revealed treatment of MV4-11 and MOLM13 cells with sitravatinib for 24 h resulted in G1 phase arrest in a dose-dependent manner (Fig. 1B, Additional file 1: Fig. S2A). Consistently, a significant increase of Annexin V positive apoptotic cells was observed in both cell lines after 48-h exposure to sitravatinib along with concurrent activation of PARP1 and caspase 8 cleavage in a dose-dependent manner (Fig. 1C, D, Additional file 1: Fig. S2B). The cell cycle blocking and pro-apoptotic effects of sitravatinib were stronger than that of gilteritinib at the same concentration, in keeping with IC50 values. Immunoblotting of MV4-11 and MOLM13 cells showed that sitravatinib-mediated anti-leukemia effects were associated with the dephosphorylation of FLT3 and its downstream molecules STAT5, AKT and ERK (Fig. 1E). We constructed BaF3-*FLT3*-ITD cells that depend on *FLT3*-ITD for survival to confirm the selective efficacy of sitravatinib on *FLT3* mutation. Sitravatinib effectively inhibited the viability of BaF3-*FLT3*-ITD cells through inactivating FLT3 signaling pathway, but did not affect the proliferation of parental BaF3 cells cultured with IL-3 (Additional file 1: Fig. S2C, D). Collectively, these results demonstrated that sitravatinib exerted potent and specific anti-leukemia effects on AML cells harboring *FLT3*-ITD mutation.

### Sitravatinib binds to FLT3 directly with high affinity

**Fig. 2:**
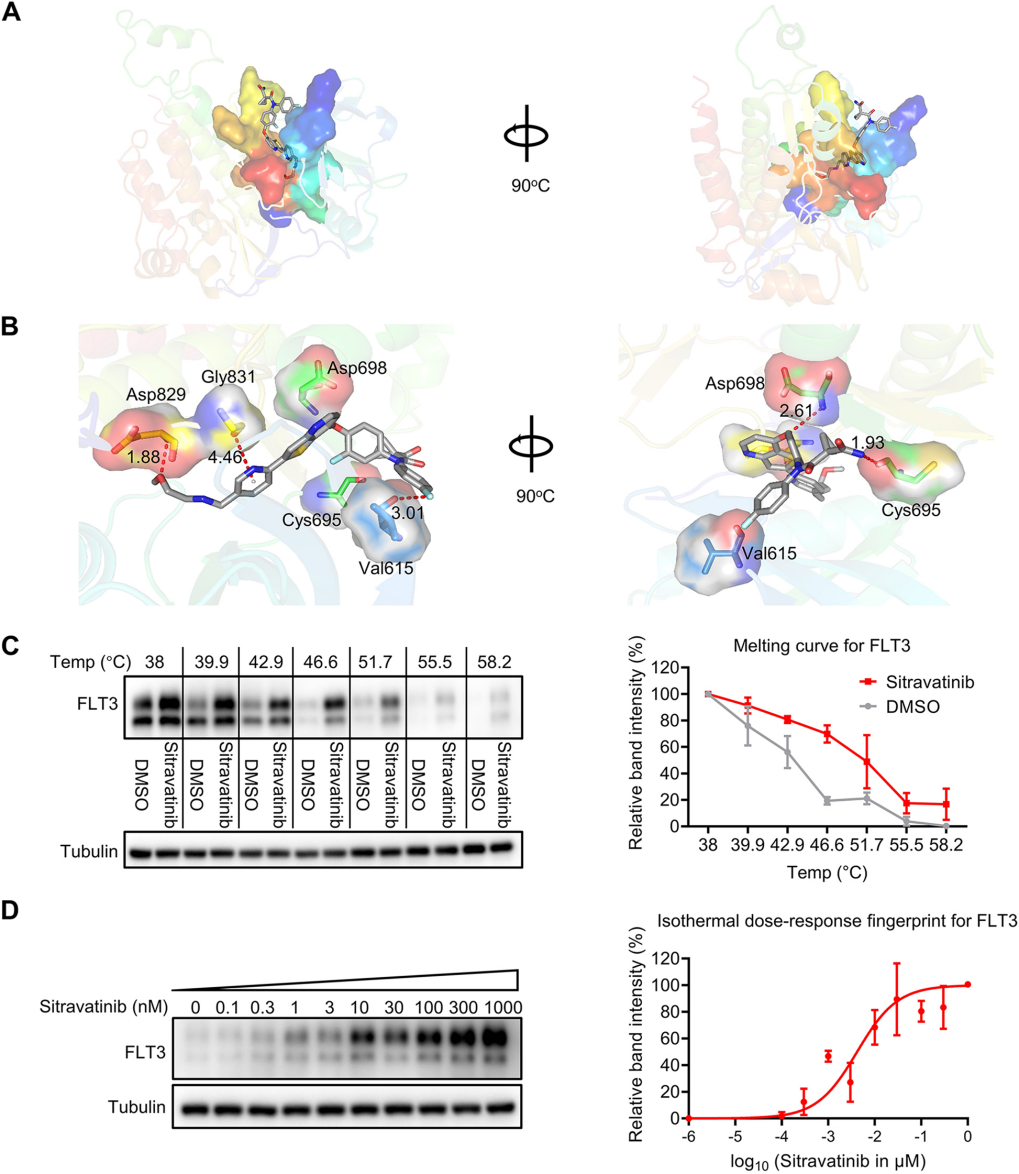
The interaction of sitravatinib and FLT3. **A** Overview of the docking results of sitravatinib with FLT3 (Protein Data Bank: 6JQR) from two orthogonal views. **B** Close-up of the sitravatinib-FLT3 model highlighted the detailed binding sites of sitravatinib with FLT3. The atoms of sitravatinib are colored by the type of element (grey: carbon; blue: nitrogen; red: oxygen; cyan, fluorine; yellow: sulfur). For clarity, the hydrogen atoms are omitted. Molecular interactions are shown as red lines. **C** BaF3-*FLT3*-ITD cells were treated with sitravatinib (30 μM/mL) or DMSO for 1 h, temperatures between 38–58.2 °C were defined to perform the test. Quantification was made using western blot to determine the melting curve. Data are mean ± standard error from three independent experiments. **D** BaF3-*FLT3*-ITD cells were treated with sitravatinib from 0 to 1000 nM for 30 min and then heated at 51.7 °C for 3 min. Protein quantification was made using western blot and the best fit of the data was monitored. Data are mean ± standard error from three independent experiments

To uncover the interaction between FLT3 and sitravatinib, we carried out virtual molecular docking of sitravatinib to the ATP binding sites of FLT3. The predictive minimal binding energy of sitravatinib and FLT3 protein was -14.18 kcal/mol, as compared to less -9.0 kcal/mol, which meant strong binding. The docking results indicated that sitravatinib occupied the ATP cavity of FLT3 well via forming three hydrogen bonds with amino acid residues Cys695, Asp698 and Asp829, the interaction distances of which were 1.93 Å, 2.61 Å and 1.88 Å, respectively. The model also showed that sitravatinib formed a halogen bond with Val615 and an amide-π interaction with Gly831, and the interaction distances were 3.01 Å and 4.46 Å, respectively (Fig. 2A, B). To confirm the interaction between FLT3-ITD and sitravatinib, CETSA was performed in BaF3-*FLT3*-ITD cells. Compared with DMSO treatment, there was an obvious thermal shift of the melting curve in the sitravatinib treated sample. We identified 51.7 °C at which a majority of FLT3 was disappeared in DMSO but was markedly detectable in sitravatinib for isothermal dose–response experiments (Fig. 2C). Sitravatinib stabilized FLT3 in a dose-dependent manner from 0.1 nM to 1000 nM, confirming that sitravatinib could bind to FLT3-ITD directly (Fig. 2D).

### Sitravatinib is effective in *FLT3*-ITD animal models

**Fig. 3:**
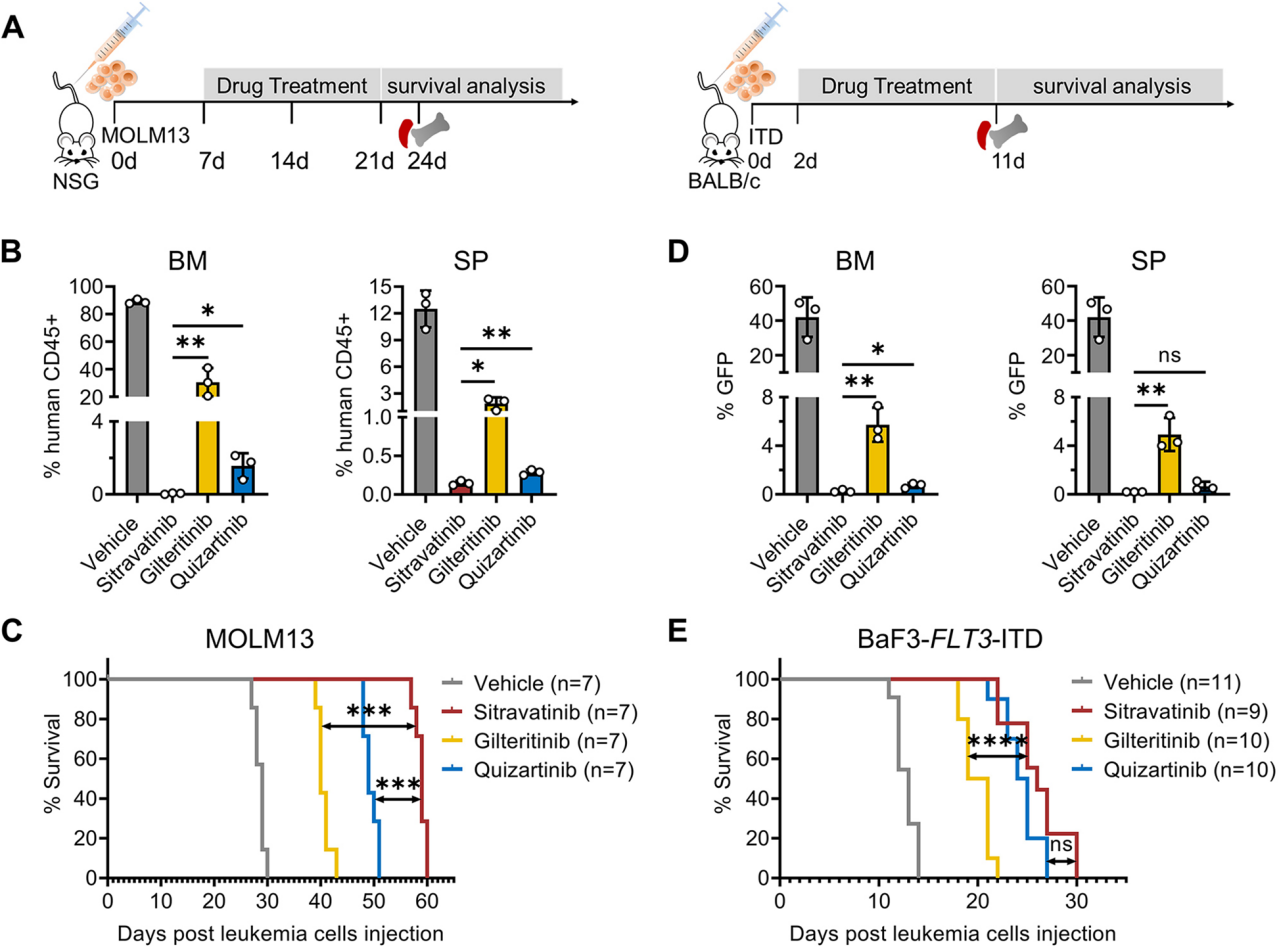
Sitravatinib suppresses leukemic progression in *FLT3*-ITD engrafted models. **A** Left: Schematic representation of xenograft experiments using human MOLM13 cells; Right: Schematic representation of transplant experiments using mouse BaF3-*FLT3*-ITD cells. **B** The percentage of human CD45 positive cells in BM and SP of MOLM13-diseased NSG mice detected by flow cytometry on day 24 (*n* = 3 mice per group). **C** The survival curves of MOLM13-diseased NSG mice treated with vehicle, sitravatinib (20 mg/kg/day), gilteritinib (30 mg/kg/day) or quizartinib (10 mg/kg/day) (*n* = 7 mice per group). **D** The percentage of GFP positive cells in BM and SP of BaF3-*FLT3*-ITD-diseased BALB/c mice on day 11 (*n* = 3 mice per group). **E** The survival curves of BaF3-*FLT3*-ITD-diseased mice treated with vehicle (n = 11), sitravatinib (20 mg/kg/day, *n* = 9), gilteritinib (30 mg/kg/day, *n* = 10) or quizartinib (10 mg/kg/day, n = 10). Error bars indicate mean ± standard error, *P < 0.05, **P < 0.01, ***P < 0.001, ****P < 0.0001

To test the in vivo antitumor efficacy of sitravatinib, we performed bone marrow xenograft experiments using MOLM13 cells. The mice were administrated vehicle, sitravatinib (20 mg/kg), gilteritinib (30 mg/kg) or quizartinib (10 mg/kg) daily for 14 consecutive days (Fig. 3A). Compared with quizartinib and gilteritinib, sitravatinib decreased the percentage of human CD45 positive cells in BM and SP to a greater extent (Fig. 3B). Vehicle-treated mice died within 30 days after injection of MOLM13 cells. Sitravatinib, gilteritinib or quizartinib treatment all prolonged the survival time of mice. Importantly, sitravatinib extended the median survival from 40 days in gilteritinib group and 49 days in quizartinib group to 59 days (Fig. 3C). We also confirmed the in vivo activity of sitravatinib using BaF3-*FLT3*-ITD mouse models. BaF3-*FLT3*-ITD cells with GFP were implanted intravenously in BALB/c mice (day 0) and drugs were administrated from the second day until the first mouse in the vehicle group died (Fig. 3A). On day 11, BM and SP samples revealed a predominance of leukemic blasts in vehicle treated mice. Mice treated with gilteritinib had a significantly lower AML burden while there were almost no leukemic cells in quizartinib and sitravatinib treated mice (Fig. 3D). Sitravatinib significantly increased survival as compared with gilteritinib, with a median survival of 26 days and 20 days, respectively (Fig. 3E). Collectively, these data support the utility of sitravatinib for treatment of *FLT3*-ITD AML.

### Gilteritinib resistant *FLT3*-ITD-F691L secondary mutation is sensitive to sitravatinib both in vitro and in vivo

**Fig. 4:**
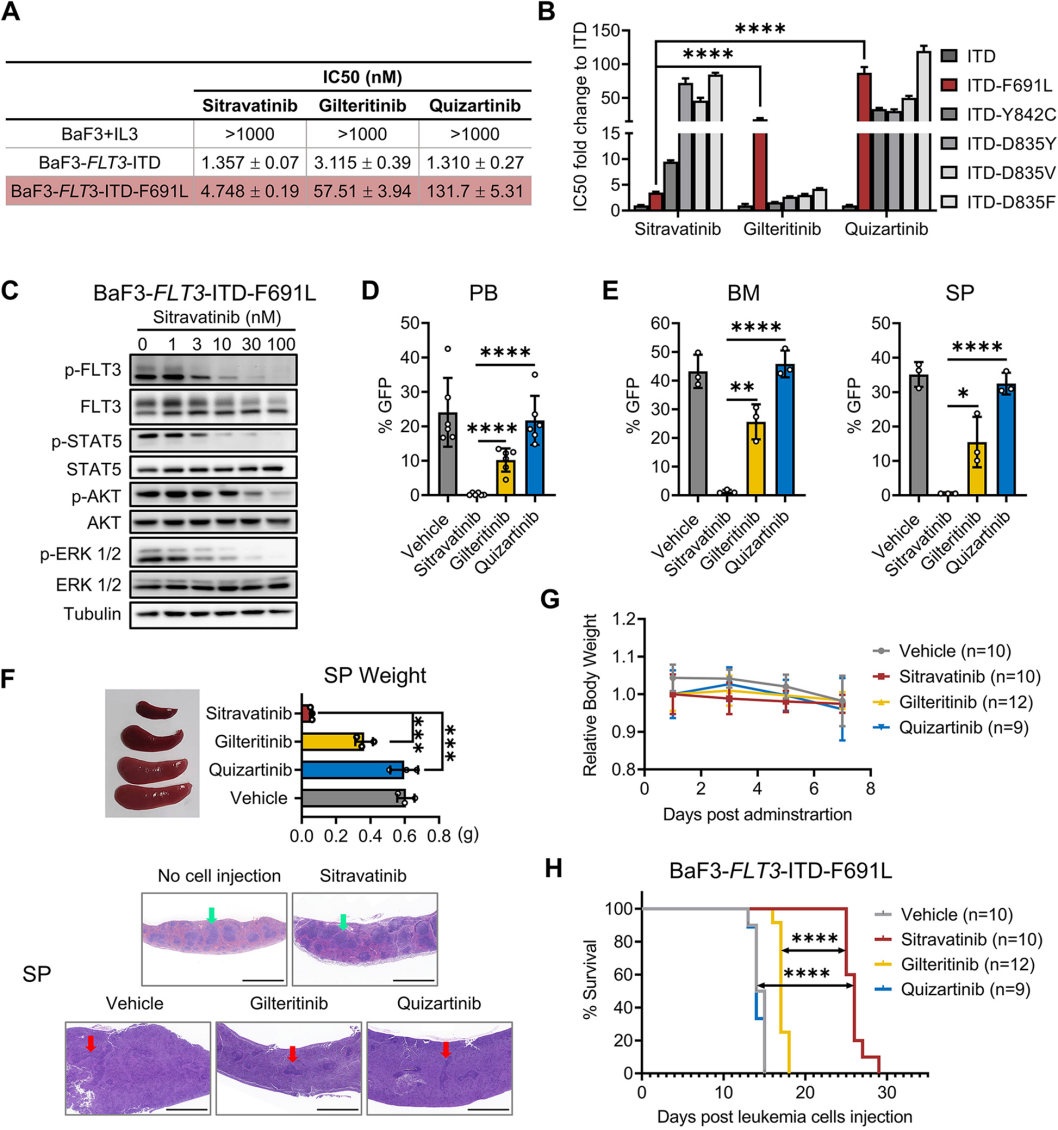
Sitravatinib is efficient to eliminate *FLT3*-ITD-F691L cells in vitro and in vivo. **A** IC50 values of BaF3-*FLT3*-ITD and BaF3-*FLT3*-ITD-F691L cells treated with each drug for 48 h. Error bars indicate mean ± standard error, *n* = 3 independent experiments. **B** Fold changes of IC50 values of FLT3 inhibitors for BaF3-*FLT3*-ITD cells with or without secondary TKD mutations. Error bars indicate mean ± standard error, *n* = 3 independent experiments. **C** Western blot analysis of p-FLT3, p-STAT5, p-AKT and p-ERK 1/2 in BaF3-*FLT3*-ITD-F691L cells after treatment with sitravatinib at the indicated concentrations for 4 h. **D** BALB/c mice were intravenously inoculated with BaF3-*FLT3*-ITD-F691L cells. From 2 days after the injection, mice were administrated with vehicle, sitravatinib (20 mg/kg/day), gilteritinib (30 mg/kg/day) or quizartinib (10 mg/kg/day) until the first mouse died in the vehicle group. The percentage of leukemia cells in PB of BaF3-*FLT3*-ITD-F691L-diseased mice on day 10 was detected by flow cytometry (*n* = 6 mice per group). **E** The percentage of leukemia cells in BM and SP of BaF3-*FLT3*-ITD-F691L-diseased mice on day 10 (*n* = 3 mice per group). **F** Top: The spleen image and weight of mice in (E); Bottom: One mouse of each group in (E) was randomly selected for the H&E staining of spleens. A normal mouse used as control. Scale bars: 1500 μm. Green arrows: typical spleen lymph nodules; red arrows: fuzzy lymph nodules. **G** Body weight measurements of the mice during drug administration. **H** The survival curves of BaF3-*FLT3*-ITD-F691L-diseased mice treated with vehicle (*n* = 10), sitravatinib (*n* = 10), gilteritinib (*n* = 12) or quizartinib (*n* = 9). Error bars indicate mean ± standard error, *P < 0.05, **P < 0.01, ***P < 0.001, ****P < 0.0001

Secondary mutation F691L develops during gilteritinib therapy as a cause of resistance and there is no applicable therapeutic option for patients with F691L mutation [34]. Structurally, gilteritinib interacted with F691 residue via van der Waals or CH − π interactions and quizartinib formed π-π interactions with F691 residue [35, 36]. Unlike gilteritinib and quizartinib, the predicted binding sites of sitravatinib with FLT3 did not include F691, indicating that sitravatinib might overcome F691L resistance. We tested the inhibitory effects of sitravatinib on a panel of BaF3 cells expressing clinically relevant *FLT3*-ITD-TKD mutants in comparison with gilteritinib and quizartinib. Consistent with previous studies, quizartinib was vulnerable to *FLT3*-ITD-D835Y/V/F, *FLT3*-ITD-Y842C and *FLT3*-ITD-F691L while gilteritinib was effective against D835 and Y842 point mutations but useless for *FLT3*-ITD-F691L (Additional file 1: Fig. S3). In contrast to gilteritinib, sitravatinib demonstrated potent inhibitory activity against *FLT3*-ITD-F691L, and to a lesser degree, inhibited *FLT3*-ITD-Y842C, but failed to curb *FLT3*-ITD-D835V/Y/F (Fig. 4A, B, Additional file 1: Fig. S3). Western blot showed the inhibitory levels of sitravatinib on FLT3 signaling pathway in BaF3-*FLT3*-ITD-TKD cells were parallel with its antiproliferation activities. Sitravatinib at a concentration of 3 nM successfully suppressed the phosphorylation of FLT3, STAT5, AKT and ERK in BaF3-*FLT3*-ITD-F691L cells. (Fig. 4C, Additional file 1: Fig. S4). To assess the in vivo activity of sitravatinib against F691L, we intravenously injected BaF3-*FLT3*-ITD-F691L into BALB/c mice (day 0). Compared to vehicle, gilteritinib and quizartinib treatment, sitravatinib significantly decreased the percentage of GFP-positive (BaF3-*FLT3*-ITD-F691L) cells in PB, BM and SP on day 10 (Fig. 4D, E). Spleen sizes were markedly reduced under sitravatinib treatment. Taking the healthy mouse without cell injection as a control, H&E staining of the spleen/liver showed a normal structure in sitravatinib treated mouse while the structural destruction and heteromorphic cell infiltration were observed in other groups (Fig. 4F, Additional file 1: Fig. S5). No obvious weight loss or any other signs of toxicity were observed among the groups during the dosing period (Fig. 4G). Sitravatinib markedly prolonged the median survival of BaF3-*FLT3*-ITD-F691L diseased mice from 14.5 days in vehicle to 26 days, while gilteritinib only extended median survival by 2.5 days than vehicle (Fig. 4H). In contrast, sitravatinib was less effective than gilteritinib in mice engrafted with BaF3-*FLT3*-ITD-Y842C cells (median survival: 16 *vs.* 20 days), but still significantly better than vehicle (*P* < 0.001) (Additional file 1: Fig. S6). In agreement with the in vitro results, sitravatinib did not have an obvious benefit on the survival of mice bearing leukemia cells harboring secondary D835V point mutations (Additional file 1: Fig. S7). Overall, our results suggest that sitravatinib is a promising second-line therapy to overcome gilteritinib resistance conferred by F691L mutation.

### Effective activity of sitravatinib against FLT3 signaling pathway in high FL/FGF2 milieu

**Fig. 5:**
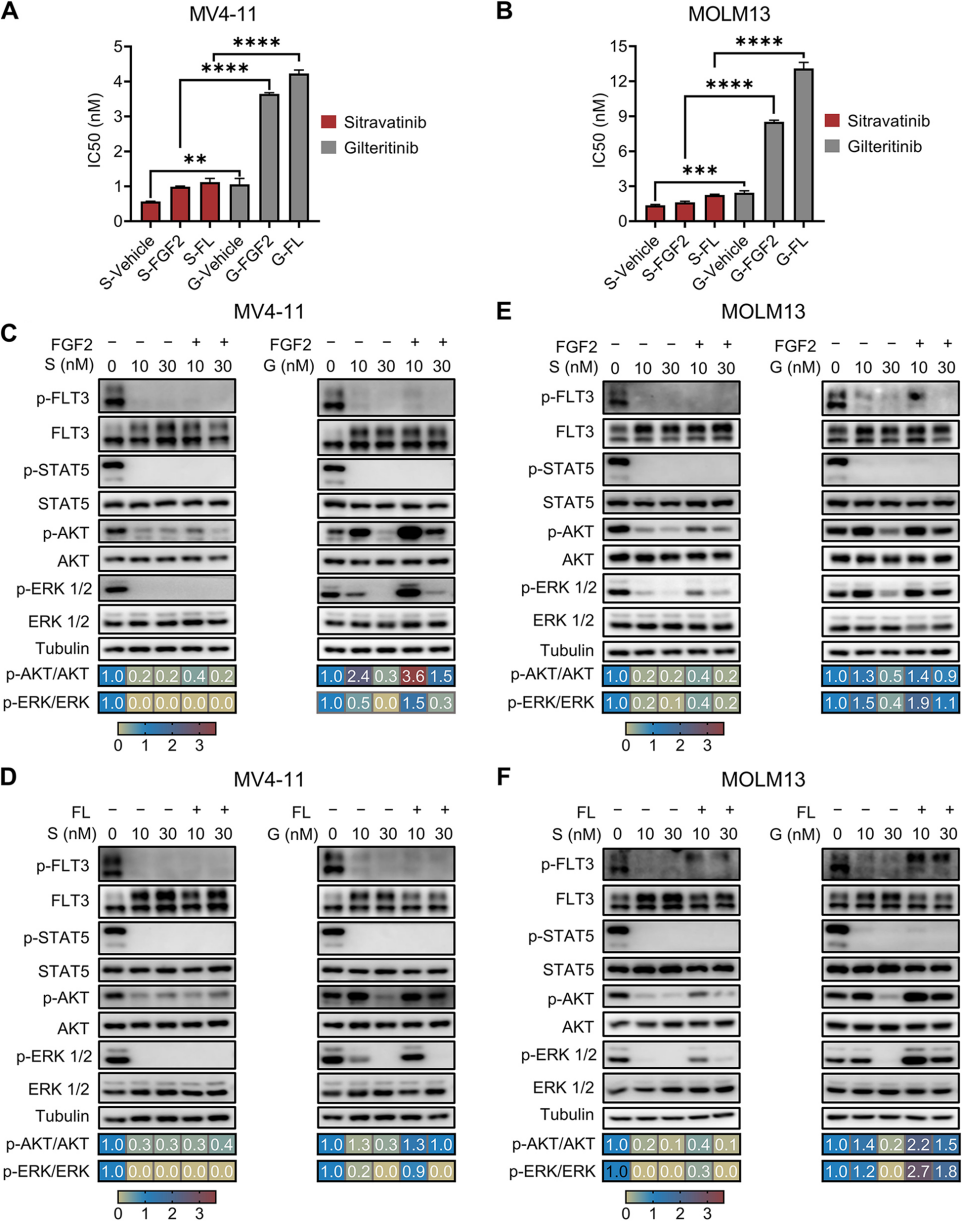
Sitravatinib efficiently suppresses the resistance mediated by FGF2 and FL. **A**-**B** The IC50 values of sitravatinib (S) and gilteritinib (G) for MV4-11 or MOLM13 cells in the absence/presence of FGF2 or FL. Error bars indicate mean ± standard error, *n* = 3 independent experiments. **P < 0.01, ***P < 0.001, ****P < 0.0001. **C**-**F** MV4-11 and MOLM13 cells were treated with sitravatinib or gilteritinib at indicated concentrations for 3 h, then FGF2 or FL (10 ng/mL) was added to the culture for incubation of 3 h. Cell lysates were immunoblotted to evaluate p-FLT3, p-STAT5, p-AKT and p-ERK 1/2. The densitometry ratio of p-AKT to AKT and p-ERK to ERK was assessed via ImageJ, and expressed as fold change compared with vehicle control

Increased levels of FGF2 and FL support the survival of residual leukemia cells during gilteritinib treatment, thus inducing early resistance and catalyzing late resistance [15]. We evaluated the cytotoxic effects of sitravatinib and gilteritinib on MOLM13 and MV4-11 cells in high FL/FGF2 milieu. Exogenous FL or FGF2 induced an upward shift in the gilteritinib dose–response curves and increased IC50 values of gilteritinib by 3.4 to 5.4 fold. However, the inhibitory effect of sitravatinib was slightly affected by the addition of FL or FGF2 with a mild increase in IC50 values (Fig. 5A, B, Additional file 1: Fig. S8). Immunoblotting results showed that sitravatinib possessed more effective activity against FLT3 downstream signaling including MAPK and AKT pathways than gilteritinib in the absence of FL/FGF2. It was notable that p-AKT and p-ERK levels rebounded under 10 nM gilteritinib treatment, which was consistent with the previous report that gilteritinib could induce ERK activation [37]. FL/FGF2 abrogated the inhibitory effect of gilteritinib on p-AKT and p-ERK. Nevertheless, sitravatinib exerted comparable activity to suppress p-AKT and p-ERK in FL/FGF2 milieu as those without cytokine treatment (Fig. 5C-F). These data suggest that sitravatinib could overcome the protective effects of FL/FGF2, which play a critical role in gilteritinib resistance, through its profound inhibition of p-ERK and p-AKT.

### Activity of sitravatinib against blasts from AML patients

**Fig. 6:**
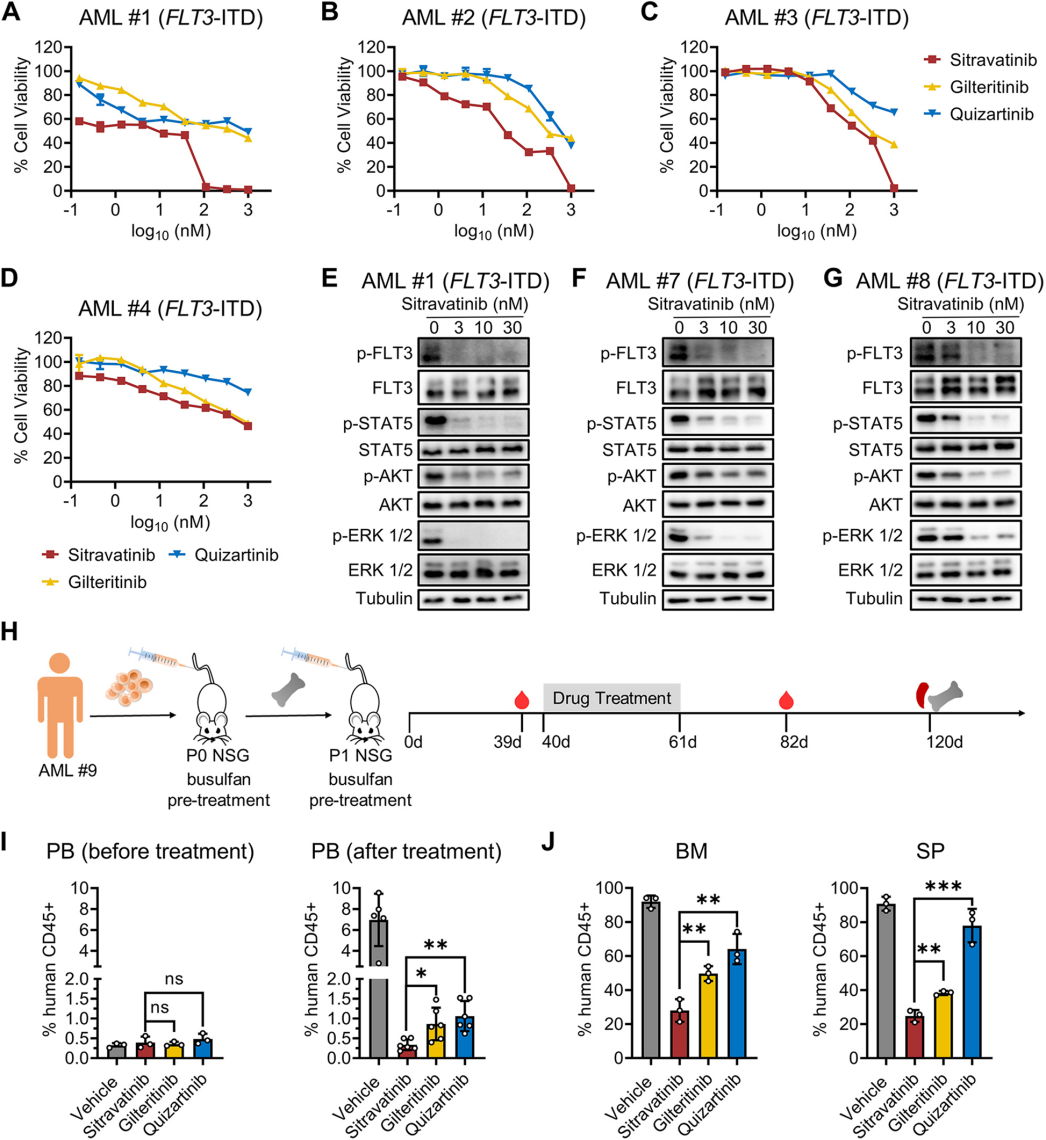
Anti-leukemia activity of sitravatinib against AML primary blasts. **A**-**D** Dose–response curves of primary AML patient samples diagnosed as *FLT3*-ITD treated with sitravatinib, gilteritinib or quizartinib at indicated concentrations for 48 h. **E**–**G** Western blot analysis of p-FLT3, p-STAT5, p-AKT, p-ERK 1/2 in AML blasts harboring *FLT3*-ITD after 6-h sitravatinib treatment. **H** Schematic representation of the PDX model experiments. **I** The percentage of human CD45 positive cells in PB of PDX mice detected by flow cytometry on day 39 (*n* = 3 mice per group) and day 82 (*n* = 5 or 6 mice per group). **J** The percentage of human CD45 positive cells in BM and SP of PDX mice detected by flow cytometry on day 120 (*n* = 3 mice per group). Error bars indicate mean ± standard error, *P < 0.05, **P < 0.01, ***P < 0.001

To examine the anti-leukemia activity of sitravatinib on primary cells, we collected bone marrow cells from 8 patients, 6 of whom were diagnosed as *FLT3*-ITD and 2 of whom expressed wild-type *FLT3*. The overall magnitude of antileukemic effect of sitravatinib on blasts harboring *FLT3*-ITD was more effective than that of quizartinib and gilteritinib regardless of the disease state (Fig. 6A-D). As expected, *FLT3* wild-type blasts were insensitive to sitravatinib as well as gilteritinib and quizartinib (Additional file 1: Fig. S9A). We also evaluated the effect of sitravatinib on PBMC from healthy donors. The results showed that the cell viability of normal blood cells exhibited no response to sitravatinib treatment, indicating the good safety of sitravatinib (Additional file 1: Fig. S9B). Western blot data showed that sitravatinib markedly reduced the levels of p-FLT3, p-STAT5, p-AKT and p-ERK in primary blasts, demonstrating that the anti-leukemia activity of sitravatinib was coupled with the suppression of FLT3 signaling pathways (Fig. 6E-G). We also utilized a *FLT3*-ITD AML PDX model to further predict the patient responses to sitravatinib treatment. Drug administration was started 40 days after transplantation and continued for 21 days (Fig. 6H). On the day before treatment, the mean percentage of AML cells in PB was similar among groups (about 0.35%). On the 21st day after finishing treatment, PB analysis showed that there were slight differences between sitravatinib and other drugs. The mean percentage of AML cells in PB in vehicle, gilteritinib, quizartinib and sitravatinib group was 6.956%, 0.863%, 1.062% and 0.33%, respectively (Fig. 6I). On the 59st days after treatment, BM and SP detection revealed that the mice treated with sitravatinib had a lower leukemia burden than those treated with gilteritinib and quizartinib (Fig. 6J). Weight loss or any other signs of toxicity were not observed in any group during treatment (Additional file 1: Fig. S9C). To further explore the potential mechanisms of sitravatinib’s therapeutic advantage, we analyzed the transcriptome of MOLM13 cells with different treatments and performed KEGG enrichment analysis. Sitravatinib down-regulated the expression level of “metabolic pathways” as compared with gilteritinib, which deserved further study (Additional file 1: Fig. S10).

## Discussion

AML is the most common hematological malignancy with high lethality, especially for the elderly people population [38]. Considering the high prevalence and adverse prognosis of *FLT3* mutations in AML, sustained efforts have been made in developing FLT3 inhibitors over the last decades [12, 39]. Among them, gilteritinib and midostaurin have received regulatory approval throughout much of the world and have been listed as the guideline recommendation [34], while quizartinib is available only in Japan. Despite dramatic improvements in clinical outcomes, current FLT3 inhibitors have considerable limitations. Midostaurin is applied in combination with 3 + 7 chemotherapy for newly diagnosed patients due to its poor performance in monotherapy, which prevents its administration in unfit elderly patients [40]. Quizartinib showed dose-dependent adverse effects, as exemplified to severe QT interval prolongation with ventricular arrhythmia events [41, 42]. Gilteritinib as the only monotherapy widely used in clinical indeed improved median overall survival than salvage chemotherapy (9.3 *vs.* 5.6 months) for refractory/relapsed patients, but approximately one third of patients had no response and only 37% of patients survived more than one year [11]. Of note, drug resistance from acquired mutations or other mechanisms remains a ubiquitous challenge for all these inhibitors and ultimately leads to disease progression in almost all patients [9, 17, 20, 43]. These clinical results indicate that new potent inhibitors with fewer toxicities to overcome current drug resistance are of unmet clinical requirement. Here, we evaluated the efficacy of sitravatinib in *FLT3* mutant AML.

Firstly, we found that sitravatinib displayed selective and potent growth inhibitory effect on *FLT3*-ITD AML cell lines in vitro. Further CETSA assays revealed that sitravatinib was a FLT3 inhibitor. In vivo studies confirmed the anti-tumor activity of sitravatinib in both MOLM13 xenograft models and BaF3-*FLT3*-ITD models, resulting in a survival benefit superior to that of gilteritinib. No obvious drug toxicity was observed during sitravatinib treatment, which was in line with the data of clinical trials in solid tumors. Taken together, these data support sitravatinib as a favorable inhibitor for *FLT3*-ITD AML.

Then, we demonstrated that sitravatinib could overcome gilteritinib resistance. On-target resistance from secondary point mutations in TKD of *FLT3*-ITD, mostly occurring at D835 and F691 represents a common mechanism responsible for FLT3 inhibitor resistance [17, 43, 44]. Most TKD mutations are vulnerable to gilteritinib but the gatekeeper mutation F691L develops during gilteritinib treatment and confers resistance to all clinical inhibitors, highlighting an urgent medical need [18, 20]. In our study, sitravatinib was proved to inhibit proliferation and aberrant signaling transduction in BaF3 cells bearing *FLT3*-ITD-F691L mutations. Remarkable therapeutic advantages were observed in *FLT3*-ITD-F691L mouse models under sitravatinib treatment. Therefore, sitravatinib offers a promising strategy for the treatment of AML patients with acquired resistance to gilteritinib or quizartinib as a consequence of secondary TKD mutation at F691. The activity of sitravatinib against F691L may be associated with its unique binding mode to FLT3, which need to be elucidated in future studies. Besides on-target resistance, bypass signaling pathways replacing FLT3 for blasts survival account for resistance against FLT3 inhibitors [9, 45–47]. Factors emitted by the bone marrow microenvironment can activate MAPK and AKT pathways to mediate resistance to FLT3 inhibitors [48, 49]. Our data showed that the efficacy of sitravatinib was less impeded by FL and FGF2 compared with gilteritinib, which was mediated through the more potent and steady inhibition of p-ERK and p-AKT. Therefore, sitravatinib could be a promising second-line therapy for AML patients who are resistant to other FLT3 inhibitors.

Among other known targets of sitravatinib, AXL has been demonstrated to support the growth of *FLT3*-ITD cells through positive regulation of constitutive FLT3 activation [21, 50]. The activation and upregulation of AXL also play a pivotal role in the development of midostaurin and quizartinib resistance [51, 52]. Previous studies showed that sitravatinib could significantly decrease AXL phosphorylation [21], which might also contribute to its efficacy against *FLT3*-ITD leukemia. Furthermore, recent studies revealed some unique characteristics of sitravatinib. Sitravatinib possesses the ability to reduce immunosuppressive myeloid cells and potentiate immune checkpoint blockade in preclinical and clinical models [22, 23]. The combination of sitravatinib and anti–programmed death-1 (PD-1) therapy has demonstrated high clinical activity with manageable toxicity in oral cavity cancer and renal cancer [22, 23]. Sitravatinib has also been shown to block the chemotherapeutic drug efflux function of ABCB1 and ABCG2 at submicromolar concentrations. In preclinical models, sitravatinib enhanced the antitumor activity of chemotherapeutic agents and reversed multidrug resistance [53, 54]. Our RNA-sequencing data indicates that sitravatinib might also regulate the metabolism of leukemia cells, which warrants additional studies. Sitravatinib has exerted remarkable antileukemic activity in patient blasts and a *FLT3*-ITD PDX model. It is hopeful to see that sitravatinib will show effective therapeutic results in AML patients harboring FLT3 mutations with its multifold effects in clinical.

## Conclusions

In summary, we report that sitravatinib is a potent FLT3 inhibitor in AML, which can overcome clinically relevant gilteritinib resistance conferred by F691L mutation and cytokines. Given the safety profile in clinical studies of solid tumors, further trials of sitravatinib in AML either singly or in combination with other modalities, as exemplified to chemotherapy and immunotherapy, are warranted in the near future.

## Supplementary Information

**Additional file 1:** **Supplementary Figure S1. **Activities of sitravatinib, gilteritinib and quizartinib against AML cell lines. (A) The mutation characteristics of the AML cell lines used. (B) Dose-response curves of AML cell lines treated with sitravatinib for 48 h. (C) Dose-response curves of AML cell lines treated with gilteritinib for 48 h. (D) Dose-response curves of AML cell lines treated with quizartinib for 48 h. For (B), (C), (D), error bars indicate mean ± standard error, *n* = 3 technical replicates for each cell line. Data shown is representative of 3 independent experiments. **Supplementary Figure S2. **Sitravatinib is effective against the *FLT3*-ITD mutation in vitro. (A) Representative flow cytometry graphs of cell cycle assays from 3 independent experiments. After treatment with various concentrations of sitravatinib (S) or gilteritinib (G) for 24 h, cell cycle distributions of MV4-11 and MOLM13 cells were analyzed with PI staining. (B) Representative flow cytometry graphs of apoptosis assays from 3 independent experiments. MV4-11 and MOLM13 cell lines were treated with indicated doses of sitravatinib or gilteritinib for 48 h. Apoptosis was detected by the Annexin V/PI assay. (C) Dose-response curves of BaF3-*FLT3*-ITD cells treated with increasing concentrations of sitravatinib for 48 h. Error bars indicate mean ± standard error, *n* = 3 technical replicates. Data shown is representative of 3 independent experiments. (D) Western blot analysis of p-FLT3, p-STAT5, p-AKT and p-ERK 1/2 in BaF3-*FLT3*-ITD cells after treatment with sitravatinib at the indicated concentrations for 4 h. **Supplementary Figure S3. **Activities of sitravatinib, gilteritinib and quizartinib against *FLT3*-ITD-TKD mutants in vitro. (A) Dose-response curves of BaF3 cells harboring *FLT3*-ITD/TKD treated with sitravatinib for 48 h. (B) Dose-response curves of BaF3 cells harboring *FLT3*-ITD/TKD treated with gilteritinib for 48 h. (C) Dose-response curves of BaF3 cells harboring *FLT3*-ITD/TKD treated with quizartinib for 48 h. (D) The IC50 values of each drug for BaF3 cells harboring *FLT3*-ITD/TKD. Data are mean ± standard error from three independent experiments. For (A), (B), (C), error bars indicate mean ± standard error, *n* = 3 technical replicates for each cell line. Data shown is representative of 3 independent experiments. **Supplementary Figure S4. **Effect of sitravatinib on FLT3 signaling pathway in BaF3-*FLT3*-ITD-TKD cells. (A) Western blot analysis of p-FLT3, p-STAT5, p-AKT and p-ERK 1/2 in BaF3-*FLT3*-ITD-Y842C cells after treatment with sitravatinib at the indicated concentrations for 4 h. (B) Western blot analysis of p-FLT3, p-STAT5, p-AKT and p-ERK 1/2 in BaF3-*FLT3*-ITD-D835Y cells after treatment with sitravatinib at the indicated concentrations for 4 h. **Supplementary Figure S5. **Sitravatinib attenuates leukemic infiltration in the spleen and liver of BaF3-*FLT3*-ITD-F691L diseased mice. (A) The H&E staining pictures of spleens (the boundary region between white pulp and red pulp) from BaF3-*FLT3*-ITD-F691L diseased mice treated with vehicle, sitravatinib, gilteritinib or quizartinib at high magnification (*n* = 1 per group). A healthy mouse used as control. Scale bars: 60 μm. Green arrows: the representative lymphocytes (small, little cytoplasm). Red arrows: the representative abnormal cells (heteromorphic, irregular nucleus). (B) The H&E staining pictures of livers from BaF3-*FLT3*-ITD-F691L diseased mice treated with vehicle, sitravatinib, gilteritinib or quizartinib at low magnification (*n* = 1 per group). A healthy mouse used as control. Scale bars: 1500 μm. (C) The H&E staining pictures of livers from BaF3-*FLT3*-ITD-F691L diseased mice treated with vehicle, sitravatinib, gilteritinib or quizartinib at high magnification (*n* = 1 per group). A healthy mouse used as control. Scale bars: 60 μm. Green arrows: the representative normal nucleuses of liver cells (large, round, clear nucleolus). Red arrows: the representative abnormal nucleuses (hyperchromatic and pleomorphic). **Supplementary Figure S6. **Sitravatinib exerts therapeutic effect on *FLT3*-ITD-Y842C in vivo. (A) Schematic representation of transplant experiments using BaF3-*FLT3*-ITD-Y842C cells. (B) The percentage of GFP positive cells in PB of BaF3-*FLT3*-ITD-Y842C-diseased BALB/c mice detected by flow cytometry on day 11 (*n* = 4-6 mice per group). (C) The survival curves of BaF3-*FLT3*-ITD-Y842C-diseased mice treated with vehicle (*n* = 7), sitravatinib (20 mg/kg/day, *n* = 8), gilteritinib (30 mg/kg/day, *n* = 7) or quizartinib (10 mg/kg/day, *n* = 7). Error bars indicate mean ± standard error. *P <0.05, ***P < 0.001. **Supplementary Figure S7. **Sitravatinib has no activity against *FLT3*-ITD-D835V in vivo. (A) Schematic representation of transplant experiments using BaF3-*FLT3*-ITD-D835V cells. (B) The percentage of GFP positive cells in PB of BaF3-*FLT3*-ITD-D835V-diseased BALB/c mice detected by flow cytometry on day 11 (*n* = 4 or 5 mice per group). (C) The survival curves of BaF3-*FLT3*-ITD-D835V-diseased mice treated with vehicle (*n *= 6), sitravatinib (20 mg/kg/day, *n* = 7), gilteritinib (30 mg/kg/day, *n* = 7) or quizartinib (10 mg/kg/day, *n* = 6). Error bars indicate mean ± standard error. *P <0.05, **P < 0.01, ****P < 0.0001. **Supplementary Figure S8. **The efficacy of sitravatinib is less affected by FGF2 and FL. (A-D) Dose-response curves of MV4-11 and MOLM13 cells in culture ± recombinant FGF2 or FL (10 ng/mL) treated with a gradient of gilteritinib or sitravatinib for 48 h. Error bars indicate mean ± standard error, *n* = 3 technical replicates for each cell line. Data shown is representative of 3 independent experiments. (E-F) Fold changes of IC50 values of sitravatinib (S) and gilteritinib (G) for MV4-11 or MOLM13 cells after the addition of FGF2 or FL. Error bars indicate mean ± standard error, *n* = 3 independent experiments. ****P< 0.0001. **Supplementary Figure S9. **Sitravatinib shows good safety. (A) Dose-response curves of primary AML patient samples diagnosed as *FLT3*-WT treated with sitravatinib, gilteritinib or quizartinib at indicated concentrations for 48 h. (B) Dose-response curves of PBMC from healthy donors after treatment with increasing concentrations of sitravatinib for 48 h. (C) Body weight measurements of the PDX model mice on day 1, 8, 15, and 21 post drug administration. Error bars indicate mean ± standard error. **Supplementary Figure S10. **KEGG enrichment of genes down-regulated by sitravatinib compared with gilteritinib. MOLM13 cells were treated with gilteritinib (10 nM) or sitravatinib (10 nM) for 24 h and then subjected to RNA-sequencing analysis. KEGG pathway enrichment was performed on genes down-regulated in sitravatinib-treated cells with absolute value of fold change ≥ 2 and *p* < 0.05 (as compared to gilteritinib-treated cells). **Supplementary Table S1. **Clinical information relevant to AML patient samples. 

## Data Availability

The datasets used and analyzed during the current study are available from the corresponding author on reasonable request.

## Abbreviations

FLT3: FMS-like tyrosine kinase 3
ITD: Internal tandem duplication
AML: Acute myeloid leukemia
TKD: Tyrosine kinase domain
TKI: Tyrosine kinase inhibitors
BM: Bone marrow
FL: FLT3 ligand
FGF2: Fibroblast growth factor 2
IC50: The 50% inhibitory concentration
CETSA: The cellular thermal shift assay
PB: Peripheral blood
SP: Spleen
PDX: Patient-derived xenografts
PBMC: Peripheral blood mononuclear cells
WT: Wild-type
