# Supplementary material for: Sitravatinib as a potent FLT3 inhibitor can overcome gilteritinib resistance in acute myeloid leukemia
Source: Biomark Res. 2023 Jan 24;11:8. doi: 10.1186/s40364-022-00447-4 (PMC9872318; doi:10.1186/s40364-022-00447-4)
Supplement: Supplementary file 1 — Additional file 1: Supplementary Figure S1. Activities of sitravatinib, gilteritinib and quizartinib against AML cell lines. (A) The mutation characteristics of the AML cell lines used. (B) Dose-response curves of AML cell lines treated with sitravatinib for 48 h. (C) Dose-response curves of AML cell lines treated with gilteritinib for 48 h. (D) Dose-response curves of AML cell lines treated with quizartinib for 48 h. For (B), (C), (D), error bars indicate mean ± standard error, n = 3 technical replicates for each cell line. Data shown is representative of 3 independent experiments. Supplementary Figure S2. Sitravatinib is effective against the FLT3-ITD mutation in vitro. (A) Representative flow cytometry graphs of cell cycle assays from 3 independent experiments. After treatment with various concentrations of sitravatinib (S) or gilteritinib (G) for 24 h, cell cycle distributions of MV4-11 and MOLM13 cells were analyzed with PI staining. (B) Representative flow cytometry graphs of apoptosis assays from 3 independent experiments. MV4-11 and MOLM13 cell lines were treated with indicated doses of sitravatinib or gilteritinib for 48 h. Apoptosis was detected by the Annexin V/PI assay. (C) Dose-response curves of BaF3-FLT3-ITD cells treated with increasing concentrations of sitravatinib for 48 h. Error bars indicate mean ± standard error, n = 3 technical replicates. Data shown is representative of 3 independent experiments. (D) Western blot analysis of p-FLT3, p-STAT5, p-AKT and p-ERK 1/2 in BaF3-FLT3-ITD cells after treatment with sitravatinib at the indicated concentrations for 4 h. Supplementary Figure S3. Activities of sitravatinib, gilteritinib and quizartinib against FLT3-ITD-TKD mutants in vitro. (A) Dose-response curves of BaF3 cells harboring FLT3-ITD/TKD treated with sitravatinib for 48 h. (B) Dose-response curves of BaF3 cells harboring FLT3-ITD/TKD treated with gilteritinib for 48 h. (C) Dose-response curves of BaF3 cells harboring FLT3-ITD/TKD treated with [file 40364_2022_447_MOESM1_ESM.docx]

**Supplementary Information for Manuscript “Sitravatinib as a potent FLT3 inhibitor can overcome gilteritinib resistance in acute myeloid leukemia”**

**Supplementary Figure S1: Activities of sitravatinib, gilteritinib and quizartinib against AML cell lines.** (A) The mutation characteristics of the AML cell lines used. (B) Dose-response curves of AML cell lines treated with sitravatinib for 48 h. (C) Dose-response curves of AML cell lines treated with gilteritinib for 48 h. (D) Dose-response curves of AML cell lines treated with quizartinib for 48 h. For (B), (C), (D), error bars indicate mean ± standard error, *n* = 3 technical replicates for each cell line. Data shown is representative of 3 independent experiments.

**Supplementary Figure S2. Sitravatinib is effective against the *FLT3*-ITD mutation in vitro.** (A) Representative flow cytometry graphs of cell cycle assays from 3 independent experiments. After treatment with various concentrations of sitravatinib (S) or gilteritinib (G) for 24 h, cell cycle distributions of MV4-11 and MOLM13 cells were analyzed with PI staining. (B) Representative flow cytometry graphs of apoptosis assays from 3 independent experiments. MV4-11 and MOLM13 cell lines were treated with indicated doses of sitravatinib or gilteritinib for 48 h. Apoptosis was detected by the Annexin V/PI assay. (C) Dose-response curves of BaF3-*FLT3*-ITD cells treated with increasing concentrations of sitravatinib for 48 h. Error bars indicate mean ± standard error, *n* = 3 technical replicates. Data shown is representative of 3 independent experiments. (D) Western blot analysis of p-FLT3, p-STAT5, p-AKT and p-ERK 1/2 in BaF3-*FLT3*-ITD cells after treatment with sitravatinib at the indicated concentrations for 4 h.

**Supplementary Figure S3. Activities of sitravatinib, gilteritinib and quizartinib against *FLT3*-ITD-TKD mutants in vitro.** (A) Dose-response curves of BaF3 cells harboring *FLT3*-ITD/TKD treated with sitravatinib for 48 h. (B) Dose-response curves of BaF3 cells harboring *FLT3*-ITD/TKD treated with gilteritinib for 48 h. (C) Dose-response curves of BaF3 cells harboring *FLT3*-ITD/TKD treated with quizartinib for 48 h. (D) The IC50 values of each drug for BaF3 cells harboring *FLT3*-ITD/TKD. Data are mean ± standard error from three independent experiments. For (A), (B), (C), error bars indicate mean ± standard error, *n* = 3 technical replicates for each cell line. Data shown is representative of 3 independent experiments.

**Supplementary Figure S4. Effect of sitravatinib on FLT3 signaling pathway in BaF3-*FLT3*-ITD-TKD cells.** (A) Western blot analysis of p-FLT3, p-STAT5, p-AKT and p-ERK 1/2 in BaF3-*FLT3*-ITD-Y842C cells after treatment with sitravatinib at the indicated concentrations for 4 h. (B) Western blot analysis of p-FLT3, p-STAT5, p-AKT and p-ERK 1/2 in BaF3-*FLT3*-ITD-D835Y cells after treatment with sitravatinib at the indicated concentrations for 4 h.


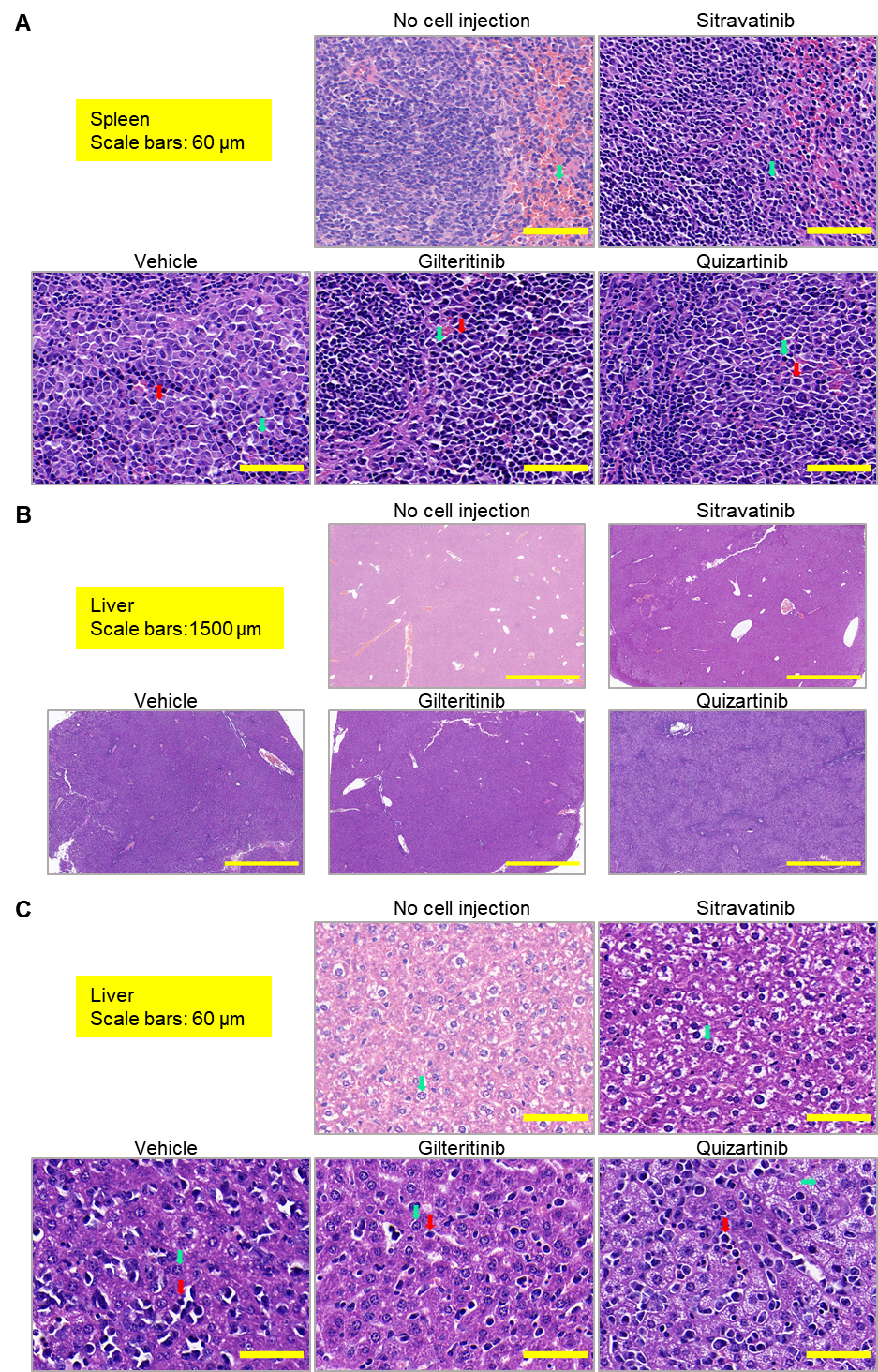


**Supplementary Figure S5. Sitravatinib attenuates leukemic infiltration in the spleen and liver of BaF3-*FLT3*-ITD-F691L diseased mice.** (A) The H&E staining pictures of spleens (the boundary region between white pulp and red pulp) from BaF3-*FLT3*-ITD-F691L diseased mice treated with vehicle, sitravatinib, gilteritinib or quizartinib at high magnification (*n* = 1 per group). A healthy mouse used as control. Scale bars: 60 μm. Green arrows: the representative lymphocytes (small, little cytoplasm). Red arrows: the representative abnormal cells (heteromorphic, irregular nucleus). (B) The H&E staining pictures of livers from BaF3-*FLT3*-ITD-F691L diseased mice treated with vehicle, sitravatinib, gilteritinib or quizartinib at low magnification (*n* = 1 per group). A healthy mouse used as control. Scale bars: 1500 μm. (C) The H&E staining pictures of livers from BaF3-*FLT3*-ITD-F691L diseased mice treated with vehicle, sitravatinib, gilteritinib or quizartinib at high magnification (*n* = 1 per group). A healthy mouse used as control. Scale bars: 60 μm. Green arrows: the representative normal nucleuses of liver cells (large, round, clear nucleolus). Red arrows: the representative abnormal nucleuses (hyperchromatic and pleomorphic).

**Supplementary Figure S6. Sitravatinib exerts therapeutic effect on *FLT3*-ITD-Y842C in vivo.** (A) Schematic representation of transplant experiments using BaF3-*FLT3*-ITD-Y842C cells. (B) The percentage of GFP positive cells in PB of BaF3-*FLT3*-ITD-Y842C-diseased BALB/c mice detected by flow cytometry on day 11 (*n* = 4-6 mice per group). (C) The survival curves of BaF3-*FLT3*-ITD-Y842C-diseased mice treated with vehicle (*n* = 7), sitravatinib (20 mg/kg/day, *n* = 8), gilteritinib (30 mg/kg/day, *n* = 7) or quizartinib (10 mg/kg/day, *n* = 7). Error bars indicate mean ± standard error. **P* < 0.05, ****P* < 0.001.

**Supplementary Figure S7. Sitravatinib has no activity against *FLT3*-ITD-D835V in vivo.** (A) Schematic representation of transplant experiments using BaF3-*FLT3*-ITD-D835V cells. (B) The percentage of GFP positive cells in PB of BaF3-*FLT3*-ITD-D835V-diseased BALB/c mice detected by flow cytometry on day 11 (*n* = 4 or 5 mice per group). (C) The survival curves of BaF3-*FLT3*-ITD-D835V-diseased mice treated with vehicle (*n* = 6), sitravatinib (20 mg/kg/day, *n* = 7), gilteritinib (30 mg/kg/day, *n* = 7) or quizartinib (10 mg/kg/day, *n* = 6). Error bars indicate mean ± standard error. **P* < 0.05, ***P* < 0.01, *****P* < 0.0001.

**Supplementary Figure S8. The efficacy of sitravatinib is less affected by FGF2 and FL.** (A-D) Dose-response curves of MV4-11 and MOLM13 cells in culture ± recombinant FGF2 or FL (10 ng/mL) treated with a gradient of gilteritinib or sitravatinib for 48 h. Error bars indicate mean ± standard error, *n* = 3 technical replicates for each cell line. Data shown is representative of 3 independent experiments. (E-F) Fold changes of IC50 values of sitravatinib (S) and gilteritinib (G) for MV4-11 or MOLM13 cells after the addition of FGF2 or FL. Error bars indicate mean ± standard error, *n* = 3 independent experiments. *****P* < 0.0001.

**Supplementary Figure S9. Sitravatinib shows good safety.** (A) Dose-response curves of primary AML patient samples diagnosed as *FLT3*-WT treated with sitravatinib, gilteritinib or quizartinib at indicated concentrations for 48 h. (B) Dose-response curves of PBMC from healthy donors after treatment with increasing concentrations of sitravatinib for 48 h. (C) Body weight measurements of the PDX model mice on day 1, 8, 15, and 21 post drug administration. Error bars indicate mean ± standard error.

**Supplementary Figure S10. KEGG enrichment of genes down-regulated by sitravatinib compared with gilteritinib.** MOLM13 cells were treated with gilteritinib (10 nM) or sitravatinib (10 nM) for 24 h and then subjected to RNA-sequencing analysis. KEGG pathway enrichment was performed on genes down-regulated in sitravatinib-treated cells with absolute value of fold change ≥ 2 and *p* < 0.05 (as compared to gilteritinib-treated cells).

| **Patient ID** | **Disease state** | **Age** | **Sex** | **Sample**  **type** | **FLT3 Status** | **Other mutations** |
| --- | --- | --- | --- | --- | --- | --- |
| AML1 | Relapsed | 44 | male | BM | *FLT3*-ITD | *BCOR, CEBPA* |
| AML2 | Untreated | 51 | male | BM | *FLT3*-ITD | *CEBPA* |
| AML3 | Untreated | 60 | male | BM | *FLT3*-ITD | *NPM1* |
| AML4 | Relapsed | 43 | female | BM | *FLT3*-ITD | *ASXL1, NF1* |
| AML5 | Untreated | 53 | male | BM | *FLT3*-WT | *DNMT3A, KRAS,*  *NRAS, NPM1* |
| AML6 | Untreated | 29 | male | BM | *FLT3*-WT | *ASXL1, CEBPA,*  *EZH2, IDH2,*  *NRAS, PHF6* |
| AML7 | Untreated | 63 | male | BM | *FLT3*-ITD | *NPM1, IDH1* |
| AML8 | Relapsed | 65 | male | BM | *FLT3*-ITD | *NPM1, DNMT3A, TET2* |
| AML9 | Untreated | 59 | female | BM | *FLT3*-ITD | *NPM1* |

**Supplementary Table S1. Clinical information relevant to AML patient samples**
